# Angiographic embolization in the treatment of arterial pelvic hemorrhage: evaluation of prognostic mortality-related factors

**DOI:** 10.1007/s00068-012-0242-6

**Published:** 2012-12-06

**Authors:** J. Lindahl, L. Handolin, T. Söderlund, M. Porras, E. Hirvensalo

**Affiliations:** 1 Department of Orthopaedics and Traumatology, Helsinki University Central Hospital, Topeliuksenkatu 5, 00260 Helsinki, Finland; 2 Department of Radiology, Helsinki University Central Hospital, Topeliuksenkatu 5, 00260 Helsinki, Finland

**Keywords:** Pelvic fracture, Acetabular fracture, Massive bleeding, Bleeding pelvic arteries, Angiographic embolization, Mortality

## Abstract

**Purpose:**

The control of arterial bleeding associated with pelvic ring and acetabular fractures (PRAF) remains a challenge for emergency trauma care. The aim of the present study was to uncover early prognostic mortality-related factors in PRAF-related arterial bleedings treated with transcatheter angiographic embolization (TAE).

**Methods:**

Forty-nine PRAF patients (46 pelvic ring and three acetabular fractures) with arterial pelvic bleeding controlled with TAE (within 24 h) were evaluated.

**Results:**

All large arterial disruptions (*n* = 7) were seen in type C pelvic ring injuries. The 30-day mortality in large vessel (iliac artery) bleeding was higher (57 %) than in medium- or small-size artery bleeding (24 %). Overall 30-day mortality was 29 %. No statistically significant difference in the first laboratory values between the survivors and nonsurvivors was found. However, after excluding patients dying of head injuries (*n* = 5), a reasonable cut-off value was identified for the base excess (BE; lower than −10 mmol/l) obtained on admission.

**Conclusions:**

PRAF patients with exsanguinating bleeding from the large pelvic artery have the worst prognosis. Very low BE values (<−10.0 mmol/l) on admission for exsanguinating patients have a negative predictive value for survival, thus anticipating a poor outcome in bleeding controlled with TAE only and an increased risk of death. In critical cases, an aggressive bleeding control protocol prompts extraperitoneal pelvic packing prior to TAE. PRAF-related rupture of the external iliac artery is rare and indicates surgical techniques in controlling and restoring blood supply to the lower leg.

## Introduction

Pelvic fractures consist of both pelvic ring and acetabular fractures (PRAF). Major pelvic injuries are associated with a high risk of venous and arterial bleeding [1, 2]. Bleeding usually originates from the presacral venous plexus, directly from the cancellous surfaces of the fracture lines, or from the main trunk or branches of iliac vessels [3–5]. In patients with blunt pelvic trauma, exsanguinating extraperitoneal bleeding remains the leading cause of early death during the first 24 h after trauma at hospital [3, 6–10].

Pelvic fracture bleeding can be temporized with emergent stabilization. Venous plexus bleeding eventually stops, as it is a low-pressure vascular system, particularly when the intra-abdominal pressure exceeds the venous pressure [11]. The results from operative ligation of the internal iliac artery have been poor [12, 13], so retroperitoneal hematoma should not be explored routinely [14]. Transcatheter angiographic embolization (TAE) has been suggested to be the method of choice for treating bleeding pelvic arteries [5, 6, 15–17]. Pelvic packing is used to control massive pelvic bleeding, and is an optional or complementary method for angiographic embolization [10, 18–22].

We have used early TAE as the main tool for controlling massive retroperitoneal hemorrhage after blunt pelvic trauma in our institution during a 12-year period. Early availability of parameters is needed in order to integrate them into the management protocol of bleeding pelvis. The aims of the present study were to evaluate the outcome after this treatment protocol (without pelvic packing), as well as to uncover early prognostic emergent parameters predisposing to treatment failure and nonsurvival of the patient.

## Materials and methods

The use of TAE in controlling acute PRAF-related bleeding was evaluated in a series of 49 consecutive patients in the Helsinki University Central Hospital Trauma Center (Töölö Hospital) between May 1996 and December 2008. Töölö Hospital is a level 1 trauma center in the Helsinki region and a tertiary referral center for severely injured patients in the southern part of Finland, with a catchment area of 1.5 million people (approximately 25 % of the Finnish population).

The data collected consisted of patient characteristics, mechanism of injury, fracture type of the pelvic ring [23] and the acetabulum [24], vital signs, additional injuries, laboratory parameters, the presence of retroperitoneal hematoma or contrast blush in trauma CT in pelvis, bleeding vessel sites and sizes in angiography, 30-day mortality, cause of death assessed by medico-legal autopsy, and TAE-associated complications. Both the first and the worst vital signs and laboratory tests at the emergency department were recorded. Fluid resuscitation and blood product replacements were recorded. Injuries were classified using the Injury Severity Score (ISS) [25] and the New Injury Severity Score (NISS) [26]. The Abbreviated Injury Scale (AIS, 2005 version) was used for ISS and NISS assessment [27].

Primary resuscitation was carried out according to the ABCDE principles. Fluid resuscitation was started with crystalloids and continued with blood component therapy. There were no major changes in the fluid resuscitation strategy during the study period, since hemostatic fluid resuscitation therapy (1:1:1) was introduced in our institute only later on. The indication for TAE was a pelvic ring and/or acetabular fracture in plain pelvic X-ray in a hemodynamically unstable patient without intra-abdominal fluid in FAST or CT, and the pelvis was considered the main source of bleeding. In cases with major free abdominal fluid, laparotomy was carried out first. External fixation of the pelvis or the pelvic binder was used as an emergency treatment prior to TAE in pelvic fractures associated with an increase in pelvic volume and/or severe (vertical) instability. The definitive surgical treatment of PRAF was carried out later, once the patient was hemodynamically stabilized.

The angiographic findings were graded as either isolated or multiple, uni- or bilateral vessel injuries. Classification of the bleeding vessel size and vessel score was performed in a similar manner to that in O’Neill et al. [3], with the vessel score being the sum of the scores of each bleeding vessel. Bleeding sites were analyzed according to the fracture type and site. Special attention was paid to the effectiveness of embolization (successful bleeding control) and re-bleeding (failure to achieve bleeding control).

Statistical comparisons of the clinical parameters were performed with SPSS 14.0 for Windows (SPSS, Inc., Chicago, IL, USA). The results are expressed as the mean ± SD for continuous unskewed variables. For skewed variables, the results are expressed as the median along with the 25th and 75th percentiles and as frequencies or percentages for categorical variables. Continuous variables between the groups were compared by the Mann–Whitney *U* test. The frequency distribution of the categorical variables was compared between the groups with the chi-squared test. The statistically significant level was set as *p* < 0.05 (two-tailed). The relationships among clinical characteristics were examined by Pearson’s correlation analysis. The study protocol was approved by the review board of the Helsinki University Central Hospital.

## Results

### Patient demographics

There were 29 males and 20 females with a mean age of 43 years (range 16–92 years; SD 21). All patients had angiography-verified arterial bleeding controlled by embolization of the bleeding vessel(s). The mean ISS and NISS were 37 ± 12 and 39 ± 11, respectively. PRAF was the only observed injury in three patients. The other most commonly injured body regions were the thorax (27 patients) and the abdomen (27 patients), followed by the extremities (23 patients). Head injury was diagnosed in 13 patients. The most common mechanisms of injury were road traffic accidents (*n* = 24) and falling from height (higher than 2 m) (*n*= 20), with the remaining cases involving major crushing (*n* = 2) and miscellaneous injury mechanisms (*n* = 3).

### Control of bleeding

The first emergency operation was laparotomy in six and lower extremity bleeding control in four patients. Three patients had laparotomy after TAE. External fixation of the pelvic ring or the pelvic binder was the primary intervention in 17 patients and the second procedure in 10 patients. One patient had symphysis plate fixation during laparotomy. Pre-angioembolization pelvic stabilization (regardless of the method used) did not correlate with survival. Seven decompressive laparotomies were carried out due to abdominal compartment syndrome occurring after TAE.

The long median interval (183 min, IQR 133–263 min) from arrival to emergency room to TAE (door to angio time) is reflected by ten patients who underwent other bleeding control procedures prior to TAE. The nonsurvivors tended to have a shorter door to angio time than the survivors (183 ± 94 vs. 283 ± 269 min), but the difference was not statistically significant (*p* = 0.27). The total average length of stay in the angio suite was 178 ± 55 min, with no difference between the survivors and nonsurvivors. Bleeding control was achieved in all of the cases except for one with a rupture of the external iliac artery, which was treated surgically after diagnosis by angiography.

### Characteristics of PRAF and bleeding arteries

There were 46 pelvic ring and three acetabular fractures. In 11 of the pelvic ring fractures, there was a concomitant acetabular fracture. The three (6 %) acetabulum fractures included two both-column fractures and one anterior column fracture with central dislocation of the femoral head. In addition, both-column acetabulum fracture patients had a concomitant type B lateral compression injury on the contralateral side. Six of the pelvic ring fractures were open fractures. Angiography revealed one bleeding artery in 21 patients (43 %) and multiple (two or more) arterial bleedings in 28 patients (57 %). In 15 patients (31 %), arterial bleeding was detected in both sides of the pelvic ring. The detailed fracture classification and the relationship between bleeding arteries (the largest bleeding artery in each patient) and mortality according to pelvic fracture type as well as an overview of the arterial injury sites are presented in Tables 1 and 2.

**Table 1:** The relationship between bleeding arteries (the largest bleeding vessel in each patient) and mortality according to the pelvic fracture type (*n* = 49)

Fracture type^a^	Patients	Arteries			Nonsurvivors
		Large^b^	Medium^c^	Small^d^	Patients
Iliac wing (type A2)	1^e^	0	1	0	1
Open book (type B1)	2	0	0	2	0
Lateral compression (type B2)	5	0	1	4	0
Unstable (type C1–C3)	38	7	16	15	11
Acetabulum	3	0	2	1	2
Totals	49	7	20	22	14

^a^Pelvic ring fracture type according to Tile’s classification and acetabular fracture type according to Judet and Letournel’s classification

^b^Large arteries: main trunk of internal or external iliac

^c^Medium-size arteries: superior gluteal, inferior gluteal

^d^Small-size arteries: iliolumbar, sacral, obturator, pudendal, vesical, and femoral circumflex

^e^92-year-old male with a comminuted type A fracture of the iliac wing

**Table 2:** Sources of arterial bleeding in 49 patients with blunt pelvic trauma

Vessel	Patients	
Internal iliac artery	42	86 %
Main trunk	6	12 %
Main branch	36	74 %
External iliac artery	2	4 %
Main trunk	1	2 %
Main branch (femoral circumflex artery)	1	2 %
Internal and external iliac arteries	5	10 %
Main trunk of internal iliac artery and femoral circumflex artery	1	2 %
Main branch of internal iliac artery and femoral circumflex artery	4	8 %

### Thirty-day mortality

The overall 30-day mortality was 29 %. Seven patients died within 24 h of admission and seven died later on (Table 3). None of the patients died due to ongoing pelvic bleeding per se. Five of the early bleeding-related deaths occurred due to the irreversible lethal triad of acidosis, hypothermia, and coagulopathy, regardless of whether the primary bleeding was controlled in the pelvic area. Three of the six patients (50 %) who underwent an emergency laparotomy died (one due to the lethal triad of acidosis, hypothermia, and coagulopathy; one due to a head injury; and one due to MOF). The overall mortality in patients with concomitant intra-abdominal bleeding necessitating laparotomy was 44 %, whereas the overall mortality in patients without intra-abdominal bleeding was 25 %.

**Table 3:** Causes of early or late death after TAE in 49 patients with blunt pelvic trauma

Patient	Age of patients	Pelvic fracture	Time of death	Cause of death
Early deaths (<24 h) *n* = 7				
1	28	C1	5 h	Head injury
2	37	BC + B2	5 h	Irreversible lethal triad^a^
3	61	C1	7 h	Irreversible lethal triad^a^
4	27	C1	7 h	Irreversible lethal triad^a^
5	66	C1	10 h	Irreversible lethal triad^a^
6	19	C1	12 h	Head injury
7	66	C3	15 h	Irreversible lethal triad^a^
Late deaths (>24 h) *n* = 7				
1	72	C1	2 days	Head injury
2	22	C1	3 days	Head injury
3	92	A2	3 days	MOF^b^
4	28	C3	3 days	Cardiac arrest^c^
5	24	C2	7 days	MOF^b^
6	74	BC + B2	11 days	Head injury
7	76	C1	22 days	MOF^b^

*BC* *+* *B2* Both-column acetabulum fracture with central dislocation of the femoral head on one side and type B lateral compression pelvic ring fracture on the other side

^a^Irreversible lethal triad of acidosis, hypothermia, and coagulopathy, regardless of whether the primary pelvic bleeding was controlled in TAE

^b^Prolonged hypovolemia-related multiple organ failure

^c^Unspecified cardiac arrest

### Survivors versus nonsurvivors

There was no statistically significant difference in age, ISS, or NISS between the survivors and nonsurvivors. There were 35 unstable type C pelvic ring fractures, and 11 out of all 14 deaths occurred in this fracture group (Table 1). There was no mortality related to type B pelvic ring injuries. In patients suffering from a fall from height, the height of the fall was significantly higher in the nonsurvivors (*n* = 6) than in the survivors (*n* = 13): 17.5 ± 5.3 versus 10.7 ± 4.5 m (*p* = 0.017).

There was no statistically significant difference in the first laboratory parameters recorded in the survivors and nonsurvivors when all patients were considered. However, after excluding the patients dying from head injuries, we found some clinically feasible parameters for identifying the nonsurvivors (Table 4). We also analyzed the results by plotting a receiver operating characteristic curve in order to obtain sensitivity and specificity values. A reasonable cut-off value was identified only for the admission BE (base excess). When a BE cut-off value of −10 mmol/l was used, the sensitivity and specificity were 75 and 62.5 %, respectively. In a later resuscitation phase, the survivors and nonsurvivors had statistically significant differences in the lowest recorded values of the parameters (Table 5).

**Table 4:** Physiological and laboratory parameters that were statistically significantly different on admission between survivors and non-survivors, except for five patients who died of head injuries (*n* = 44)

	Survivors	Nonsurvivors	*p* value
	*n* = 35	*n* = 9	
ER BE 1, mmol/l	−6.9 ± 4.5	−13.7 ± 7.9	0.024
ER platelets 1	188 ± 71	127 ± 64	0.042
ER TT % 1	62 ± 23	44 ± 18	0.038

Results are the mean ± SD for continuous, unskewed parameters

*ER* emergency room, *BE* base excess, *1* the first measured value, *TT %* thrombin time

**Table 5:** Statistically significant differences in the parameter values obtained before (ER) and after TAE between survivors and nonsurvivors (*n* = 49)

	Survivors	Nonsurvivors	*p* value
	*n* = 35	*n* = 14	
	M23/F12	M6/F8	
ER SBP w (mmHg)	83 ± 23	63 ± 23	0.021
ER pH w	7.25 ± 0.08	7.12 ± 0.16	0.008
ER BE w (mmol/l)	−7.91 ± 4.7	−14.53 ± 7.55	0.008
ER platelets w	100 ± 56	57 ± 41	0.013
ER PRBC (units)	9 ± 5.9	15 ± 8.5	0.030
Post-TAE PRBC (units)	2 (2–9.5)	10.5 (5–28.5)	0.018
Post-TAE platelets	8 (8–15)	14 (11–41)	0.031
Total PBRC (units)	26.5 (14.5–46.5)	60.5 (32.8–83.8)	0.016

Results are expressed as the mean ± SD for continuous unskewed parameters and as the median (and interquartiles) for skewed parameters

*ER* emergency room, *SBP* systolic blood pressure, *w* the worst value, *pH* acid concentration, *BE* base excess, *PRBC* packed red blood cells

### Bleeding arteries and outcome

All large arterial disruptions (*n* = 7) were seen in type C pelvic ring injuries (Table 1). Patients with a large vessel injury (internal or external iliac artery) had a significantly lower BE on ER admission than the patients with medium- or small-size vessel injuries (−14.0 ± 5.9 versus −7.2 ± 5.3, respectively; *p* = 0.011). The values of other parameters on admission did not reach statistical significance when comparing these two patient groups. Four of the seven patients with a large vessel injury died (mortality 57 %) compared to 10/42 (24 %) of those with a medium- or small-vessel injury. The difference did not reach statistical significance (*p* = 0.07), which could be due to the low number of patients (type II error). The bleeding vessel size scores were significantly higher for the nonsurvivors than the survivors (*p* = 0.004). This was also seen after omitting the nonsurvivors who died due to head injury (*p* = 0.020).

### Complications

The overall angioembolization-related complication rate was low. In one case, the signs of late bleeding prompted a repeat angiography, which revealed a new bleeding artery not seen in the first angiography. Unilateral necrosis of the gluteal muscle was seen in one patient. The etiology of ischemia of the gluteal muscle in this case was either the embolization of the internal iliac artery or direct impact and compartment syndrome in the buttock region. One patient had hemorrhage at the puncture site, which was treated with direct saturation.

## Discussion

In the present study, the maximum size of the bleeding pelvic artery and the bleeding vessel score were associated with higher mortality. The prognosis was worse amongst patients bleeding from the main trunk of the internal or external iliac artery (mortality 57 %) compared to patients bleeding from more distal and smaller arteries (mortality 24 %). Similar to our results, O‘Neill et al. [3] reported bilateral arterial bleeding in one-third of their patients. In post-mortem angiography, bilateral bleeding was seen in 17 out of 27 (64 %) accident victims [1].

In our series, 14 % of the patients had arterial bleeding from the external iliac artery or its main branch, the femoral circumflex artery. Angiography offers a good tool to identify these rare bleeding sources from the external iliac artery. All bleeding from the femoral circumflex artery (six patients) was treated successfully with selective embolization. On the other hand, rupture of the main trunk of the external iliac artery was the only arterial injury prompting immediate surgical repair.

Both the pelvic external fixator and circumferential compression devices have been reported to be effective in the early stabilization of unstable pelvic injuries [11, 28–30]. In the present series, the external fixator or the pelvic binder was used as an emergency procedure in pelvic fractures associated with an increase in the pelvic volume and/or severe vertical instability. However, we did not see any correlation with the survival, which may be biased due to the low number of patients. It should also be kept in mind that our series did not reveal all bleeding sites that may be due to venous injuries. The use of the pelvic binder is part of the routine protocol in our clinic, but the binder is applied only after the plain pelvic X-ray is taken, since lateral compression may cause further bleeding in acetabular fractures with central dislocation of the femoral head or in type B lateral compression injuries with severe dislocation in a rami fracture site or in comminuted iliac wing fractures (the use of compression is contraindicated).

Pelvic arterial hemorrhage due to an acetabulum fracture is considered to be very rare. In the present study, we had three acetabular fractures with arterial pelvic bleeding (6.1 %). In all of these three fractures, the femoral head was displaced centrally as an injury associated with anterior column or both-column acetabular fractures. These findings are similar to those described in earlier reports of acetabular fracture-related arterial bleeding, in terms of prevalence [5] and fracture morphology [31, 32].

Eleven out of the 14 nonsurvivors in the present study had an unstable type C pelvic ring injury. This was in conjunction with the finding that all except four injuries of the large- and medium-size arteries were type C pelvic ring injuries. However, we were not able to find statistically significant differences in the pelvic/acetabular fracture type between the survivors and nonsurvivors. This may be due to a relatively low number of patients and certain fracture types in the present study that lead to type II error, resulting in missing something that actually was present.

Several factors have been found to predict major bleeding and mortality in patients with blunt pelvic trauma: SBP <90 mmHg [3, 8, 33, 34], low hemoglobin level [8], high blood lactate level [35], the age of the patient [7, 9, 33, 34, 36], ISS [34, 36–38], and RTS (Revised Trauma Score) [33, 36]. However, none of these parameters is specific enough to uncover patients at the highest risk of death. We found some clinically feasible parameters for identifying nonsurvivors amongst massively bleeding blunt pelvic trauma patients after the exclusion of patients dying of head injuries. A reasonable cut-off value was only identified for BE obtained on admission (the first recorded BE). When using a BE cut-off value of less than −10 mmol/l, we could identify 75 % of the pelvic arterial bleeders who would not survive. On the other hand, the specificity for that was relatively low, meaning that 37.5 % of the patients with a BE of less than −10 still survived. Our finding is in accordance with that of Starr et al. [33], who reported that a BE of less than −10.1 predicted mortality with a sensitivity of 33 % and specificity of 91 %.

Angiographic embolization should be considered without delay in patients with pelvic injuries who remain hemodynamically unstable, regardless of the occurrence of adequate resuscitation and provisional stabilization of the pelvic ring. However, the worst prognosis is related to exsanguinating bleeding from the large pelvic artery or from multiple arteries. In the present study, the total length of stay in the angio suite was nearly 3 h on average. Although we did not see any difference between the survivors and nonsurvivors, this is an excessive period of time for a bleeding control procedure in the most critically ill patients. Thus, in a critical situation with several bleeding vessels uni- or bilaterally, it is reasonable to embolize the main trunk of the internal iliac artery to gain emergent bleeding control promptly.

In exsanguinating patients in extremis, TAE is too time-consuming, and is not a tool for traditional damage control. In these rare cases, extraperitoneal pelvic packing (EPP) may slow down the bleeding and buy the time needed to resuscitate the patient so that they can tolerate the definitive arterial bleeding control obtained by TAE [10, 18–22, 35, 39, 40]. The worsening of the lethal trial of acidosis, hypothermia, and coagulopathy results in an irreversible situation leading to death, regardless whether primary bleeding is controlled, if the period of tissue hypoperfusion becomes too long. There were five such early deaths in our series, and the question of whether some of these deaths could have been avoided with a more aggressive bleeding control protocol of emergent EPP followed by prompt nonselective iliac artery embolization remains. Consequently, we have included EPP in our latest protocol.

The major weaknesses of the present study are its retrospective nature and selected patient material (all of the patients had arterial bleeding that had already been seen in angiography), so it did not include all applicable patients with an unstable pelvic ring fracture with hemodynamic instability. The strength of our study is that consecutive patients were treated with an unchanged treatment algorithm during a long study period with only one exception: the use of trauma CT was initiated during the early years of the study period. The relatively low number of patients included in the study may lead to type II error, which must be kept in mind when interpreting our results.

We conclude that the most severe pelvic arterial bleeding is mainly related to the trunk of the internal iliac artery or its main branches, and results from a high-energy type C pelvic ring fracture. Very low BE values (less than −10.0 mmol/l) in exsanguinating patients on admission have a negative predictive value for survival, thus anticipating a poor outcome of bleeding control with TAE only and an increased risk of death. The TAE facility must be well prepared and trained if used for acute bleeding control, since bleeding control must be obtained within the first 1–2 h after the admission of such patients. In extreme cases, an aggressive bleeding control protocol of damage control EPP followed by prompt nonselective iliac artery embolization should be used.

## References

[1] Huittinen V-M, Slätis P (1973). Postmortem angiography and dissection of the hypogastric artery in pelvic fractures. Surgery.

[2] Blackmore CC, Cummings P, Jurkovich GJ, Linnau KF, Hoffer EK, Rivara FP (2006). Predicting major hemorrhage in patients with pelvic fracture. J Trauma.

[3] O’Neill PA, Riina J, Sclafani S, Tornetta P (1996). Angiographic findings in pelvic fractures. Clin Orthop Relat Res.

[4] Kataoka Y, Maekawa K, Nishimaki H, Yamamoto S, Soma K (2005). Iliac vein injuries in hemodynamically unstable patients with pelvic fracture caused by blunt trauma. J Trauma.

[5] Tötterman A, Dormagen JB, Madsen JE, Klow NE, Skaga NO, Roise O (2006). A protocol for angiographic embolization in exsanguinating pelvic trauma. A report on 31 patients. Acta Orthop.

[6] Margolies MN, Ring ED, Waltman AC, Kerr WS, Baum S (1972). Arteriography in the management of hemorrhage from pelvic fractures. New Engl J Med.

[7] Rothenberger DA, Fischer RP, Strate RG, Velasco R, Perry JF (1978). The mortality associated with pelvic fractures. Surgery.

[8] Gilliland MD, Ward RE, Barton RM, Miller PW, Duke JH (1982). Factors affecting mortality in pelvic fractures. J Trauma.

[9] Ertel W, Eid K, Keel M, Trentz O (2000). Therapeutical strategies and outcome of polytraumatized patients with pelvic injuries. A six-year experience. Eur J Trauma.

[10] Papakostidis C, Giannoudis PV (2009). Pelvic ring injuries with haemodynamic instability: efficacy of pelvic packing, a systematic review. Injury..

[11] Henry SM, Tornetta P, Scalea TM (1997). Damage control for devastating pelvic and extremity injuries. Surg Clin North Am.

[12] Hawkins L, Pomerantz M, Eiseman B (1970). Laparotomy at the time of pelvic fracture. J Trauma.

[13] Scalea TM, Sclafani S (2001). Interventional techniques in vascular trauma. Surg Clin North Am.

[14] Demetriades D, Murray JA, Asensio JA, Rich NM, Mattox KL, Hirshberg A (2004). Iliac vessel injuries. Vascular trauma.

[15] Panetta T, Sclafani SJA, Goldstein AS, Phillips TF, Shaftan GW (1985). Percutaneous transcatheter embolization for massive bleeding from pelvic fractures. J Trauma.

[16] Hakala P, Lindahl J, Alberty A, Tanskanen P, Nieminen H, Porras M (1998). Massive transfusion exceeding 150 units of packed red cells during the first 15 hours after injury. J Trauma.

[17] Cook RE, Keating JF, Gillespie I (2002). The role of angiography in the management of haemorrhage from major fractures of the pelvis. JBJS.

[18] Riska EB, von Bonsdorff H, Hakkinen S, Jaroma H, Kiviluoto O, Paavilainen T (1979). Operative control of massive haemorrhage in comminuted pelvic fractures. Int Orthop.

[19] Smith WR, Moore EE, Osborn P, Agudelo JF, Morgan SJ, Parekh AA, Cothren C (2005). Retroperitoneal packing as a resuscitation technique for hemodynamically unstable patients with pelvic fractures: report of two representative cases and a description of technique. J Trauma.

[20] Tötterman A, Madsen JE, Skaga NO, Roise O (2007). Extraperitoneal pelvic packing: a salvage procedure to control massive traumatic pelvic hemorrhage. J Trauma.

[21] Osborn PM, Smith WR, Moore EE, Cothren CC, Morgan SJ, Williams AE, Stahel PF (2009). Direct retroperitoneal pelvic packing versus pelvic angiography: a comparison of two management protocols for haemodynamically unstable pelvic fractures. Injury..

[22] Suzuki T, Smith WR, Moore EE (2009). Pelvic packing or angiography: competitive or complementary?. Injury.

[23] Tile M (1995). Fractures of the pelvis and acetabulum.

[24] Judet R, Judet J, Letournel E (1964). Fractures of the acetabulum. Classification and surgical approaches for open reduction. Preliminary report. J Bone Joint Surg Am.

[25] Baker SP, O′Neill B, Haddon W, Long W (1974). The injury severity score: a method for describing patients with multiple injuries and evaluating emergency care. J Trauma.

[26] Osler T, Baker SP, Long W (1997). A modification of the injury severity score that both improves accuracy and simplifies scoring. J Trauma.

[27] Association for the Advancement of Automotive Medicine. The Abbreviated Injury Scale 2005. http://www.aaam.org.

[28] Riemer BL, Butterfield SL, Diamond DL, Young JC, Raves JJ, Cottington E, Kislan K (1993). Acute mortality associated with injuries to the pelvic ring: the role of early patient mobilization and external fixation. J Trauma.

[29] Ghanayem AJ, Stover MD, Goldstein JA, Bellon E, Wilber JH (1995). Emergent treatment of pelvic fractures. Clin Orthop Relat Res.

[30] Spanjersberg WR, Knops SP, Schep NWL (2009). Effectiveness and complications of pelvic circumferential compression devices in patients with unstable pelvic fractures: a systematic review of literature. Injury.

[31] Stephen DJG, Kreder HJ, Day AC, McKee MD, Schemitsch EH, ElMaraghy A, Hamilton P, McLellan B (1999). Early detection of arterial bleeding in acute pelvic trauma. J Trauma.

[32] Magnussen RA, Tressler MA, Obremskey WT, Kregor PJ (2007). Predicting blood loss in isolated pelvic and acetabular high-energy trauma. J Orthop Trauma.

[33] Starr AJ, Griffin DR, Reinert CM, Frawley WH, Walker J, Whitlock SN, Borer DS, Rao AV, Jones AL (2002). Pelvic ring disruptions: prediction of associated injuries, transfusion requirement, pelvic arteriography, complications, and mortality. J Orthop Trauma.

[34] Sharma OP, Oswanski MF, Rabbi J, Georgiadis GM, Lauer SK, Stombaugh HA (2008). Pelvic fracture risk assessment on admission. Am Surg.

[35] Ertel W, Keel M, Eid K, Platz A, Trentz O (2001). Control of severe hemorrhage using C-clamp and pelvic packing in multiply injured patients with pelvic ring disruption. J Orthop Trauma.

[36] Smith W, Williams A, Agudelo J, Shannon M, Morgan S, Stahel P, Moore E (2007). Early predictors of mortality in hemodynamically unstable pelvic fractures. J Orthop Trauma.

[37] Evers BM, Cryer HM, Miller FB (1989). Pelvic fracture hemorrhage. Priorities in management. Arch Surg.

[38] Lunsjo K, Tadros A, Hauggaard A, Blomgren R, Kopke J, Abu-Zidan FM (2007). Associated injuries and not fracture instability predict mortality in pelvic fractures: a prospective study of 100 patients. J Trauma.

[39] Pohlemann T, Gännslen A, Bosch U, Tscherne H (1995). The technique of packing for control of hemorrhage in complex pelvic fractures. Tech Orthop.

[40] Gaarder C, Naess PA, Christensen EF, Hakala P, Handolin L, Heier HE, Ivancev K, Johansson P, Leppäniemi A, Lippert F, Lossius HM, Opdahl H, Pillgram-Larsen J, Røise O, Skaga NO, Søreide E, Stensballe J, Tønnessen E, Töttermann A, Örtenwall P, Östlund A. Scandinavian guidelines —the massively bleeding patient. Scand J Surg. 2008;97:15–36. 10.1177/145749690809700104 18450203

